# Transformer-based optical attenuation compensation and denoising in photoacoustic imaging

**DOI:** 10.1117/1.JBO.30.11.116004

**Published:** 2025-11-26

**Authors:** Cristian Perez Jensen, Navchetan Awasthi, Kalloor Joseph Francis

**Affiliations:** a University of Amsterdam, Informatics Institute, Faculty of Science, Mathematics and Computer Science, Amsterdam, The Netherlands; b Amsterdam UMC, Department of Biomedical Engineering and Physics, Amsterdam, The Netherlands; c Erasmus MC, Cardiovascular Institute, Department of Cardiology, Biomedical Engineering, Rotterdam, The Netherlands

**Keywords:** photoacoustic imaging, deep learning models, Transformer U-Net, fluence compensation, image quality enhancement, tumor imaging

## Abstract

**Significance:**

Linear-array-based photoacoustic imaging (PAI) combines functional imaging with structural imaging from ultrasound. However, it suffers from depth-dependent optical attenuation due to surface illumination, resulting in decreased signal amplitude and image contrast with depth. Existing attenuation compensation methods often amplify noise, creating a trade-off between depth enhancement and image quality.

**Aim:**

We aim to develop a deep learning method that addresses the coupled problem of optical attenuation compensation and denoising for linear-array-based PAI and test the applicability *in vivo*.

**Approach:**

We propose a vision-transformer-based generative model to address this coupled problem. A diverse dataset was created using simulated data and experimental twin phantoms. Vascular twin phantoms were made by printing digital images onto polyurethane films to test the performance of the model. We trained and compared three deep learning architectures, Pix2Pix, Residual U-Net, and the proposed Transformer U-Net, using various loss functions, including adversarial, MSE, PSNR, SSIM, and a combined PSNR + SSIM. We tested the model on small animal tumor images.

**Results:**

Quantitative evaluation shows that PSNR + SSIM loss is robust in preserving structural details and suppressing noise. Under the pre-specified SSIM + PSNR training objective, Trans U-Net achieves the highest SSIM and PSNR across noise levels on both datasets. *In vivo* validation using murine breast tumor models and *in vivo* breast imaging confirmed the model’s ability to enhance visualization of deep vascular structures without introducing noise amplification.

**Conclusions:**

The proposed Trans U-Net effectively addresses the coupled problem of attenuation correction and denoising in handheld PAI. This method improves depth-resolved vascular imaging and is potentially useful in clinical and preclinical photoacoustic applications.

## 1 Introduction

Photoacoustic imaging (PAI) is a promising biomedical imaging modality that combines optical contrast with ultrasonic spatial resolution.^1^ When integrated with conventional ultrasound systems, PAI enhances functional imaging capabilities, making it valuable for clinical applications such as cancer diagnosis, vascular imaging, and functional monitoring, including oxygen saturation.^2^ The probe configuration using a linear ultrasound array with an optical source is particularly popular due to its compatibility with existing ultrasound systems.^3^^–^^5^ Despite its potential, PAI with conventional ultrasound imaging systems faces significant challenges, including optical attenuation, limited target view, and the band-limited nature of the conventional linear transducers, all of which contribute to degraded image quality.^6^

Depth-dependent attenuation in PAI degrades deep-tissue contrast with the exponential decay of optical fluence. A common approach is to assume average tissue optical properties and invert fluence decay via the Beer–Lambert law, the diffusion approximation, or Monte Carlo simulations. In early work, Zhao et al.^7^ employed Monte Carlo–based light-transport simulations to generate pixel-wise fluence maps for real-time amplitude correction in a handheld probe. Although these model-based schemes enhance deep-tissue contrast, they amplify noise at greater depths.^7^^–^^9^ Hardware-augmented strategies combining diffuse optical tomography,^10^ acousto-optic tagging,^11^ or multi-illumination approaches^12^ can obtain a fluence map but require complex systems, larger probes, and computationally intensive inversions, limiting their clinical translation. Spectroscopic methods integrate wavelength-dependent diffusion models with reference-phantom calibration to provide real-time fluence correction alongside motion compensation.^12^^,^^13^ Learning-based frameworks have recently emerged as an alternative by training deep networks to perform simultaneous depth-dependent optical attenuation correction and denoising in an end-to-end fashion.^14^ Implementations using U-Nets (including residual and transformer variants), Pix2Pix GANs, and attention mechanisms have demonstrated marked improvements across simulated, phantom, and *in vivo* breast and brain imaging studies.^15^^–^^18^ Several deep-learning architectures have been developed for photoacoustic fluence compensation and optical parameter estimation, including the Residual U-Net by Cai et al.,^19^ the deep residual recurrent U-Net (DR2U-Net) by Chang et al.,^20^ O-Net by Luke et al.,^21^ EDA-Net by Yang et al.,^22^ and various encoder–decoder combinations.^23^ Most prior deep-learning methods for PAI focus on denoising, contrast enhancement, or quantitative oxygenation mapping. Although effective in those tasks, they generally do not address depth-dependent optical attenuation in tomographic and handheld linear-array systems. Further, these works do not address the optical attenuation correction and denoising as a coupled problem. Our work fills this gap by introducing a transformer-based model that jointly compensates attenuation and suppresses noise, enabling robust depth-resolved vascular imaging.

In this work, we address the challenge of depth-dependent optical attenuation and noise amplification in PAI as a joint problem, using a vision transformer (ViT)-based Trans U-Net. To train and test the models, we developed a comprehensive dataset that bridges simulation and experiment. It includes: (1) simulated photoacoustic images using combined optical and acoustic modeling, with uniform fluence images as ground truth and their counterparts incorporating realistic light propagation in tissue-mimicking media; (2) experimental images of printed phantoms acquired in water as a non-scattering medium as reference images and in tissue-mimicking scattering phantoms, capturing real-world complexity of imaging using a handheld probe. By utilizing the same digital phantoms and recreating them through printing, we created “digital and experimental twins,” enabling controlled validation against known ground truth. We systematically evaluated a set of conditional generative models, Pix2Pix, lightweight Residual U-Net (Res18), and also proposed a Transformer U-Net model, across different measurement noise levels and experimental setups, including *in vivo* data. We also explored multiple loss functions, including adversarial loss, mean squared error (MSE), peak signal-to-noise ratio (PSNR), structural similarity index (SSIM), and a combined PSNR + SSIM formulation. Finally, we test the proposed model on small animal tumor imaging.

## 2 Methods

We used two vascular datasets and optical simulations to model light propagation, followed by acoustic simulations and image reconstruction for our PAI dataset. We printed these images onto polyurethane films and imaged them with our PAI system to create experimental datasets. We generated both ideal and realistic PAI for model training and testing, and evaluated deep learning models and loss outputs with various image quality metrics. Subsequent sections provide details about the dataset generation, model training, and image evaluation.

### 2.1 Dataset Generation

**Fig. 1:**
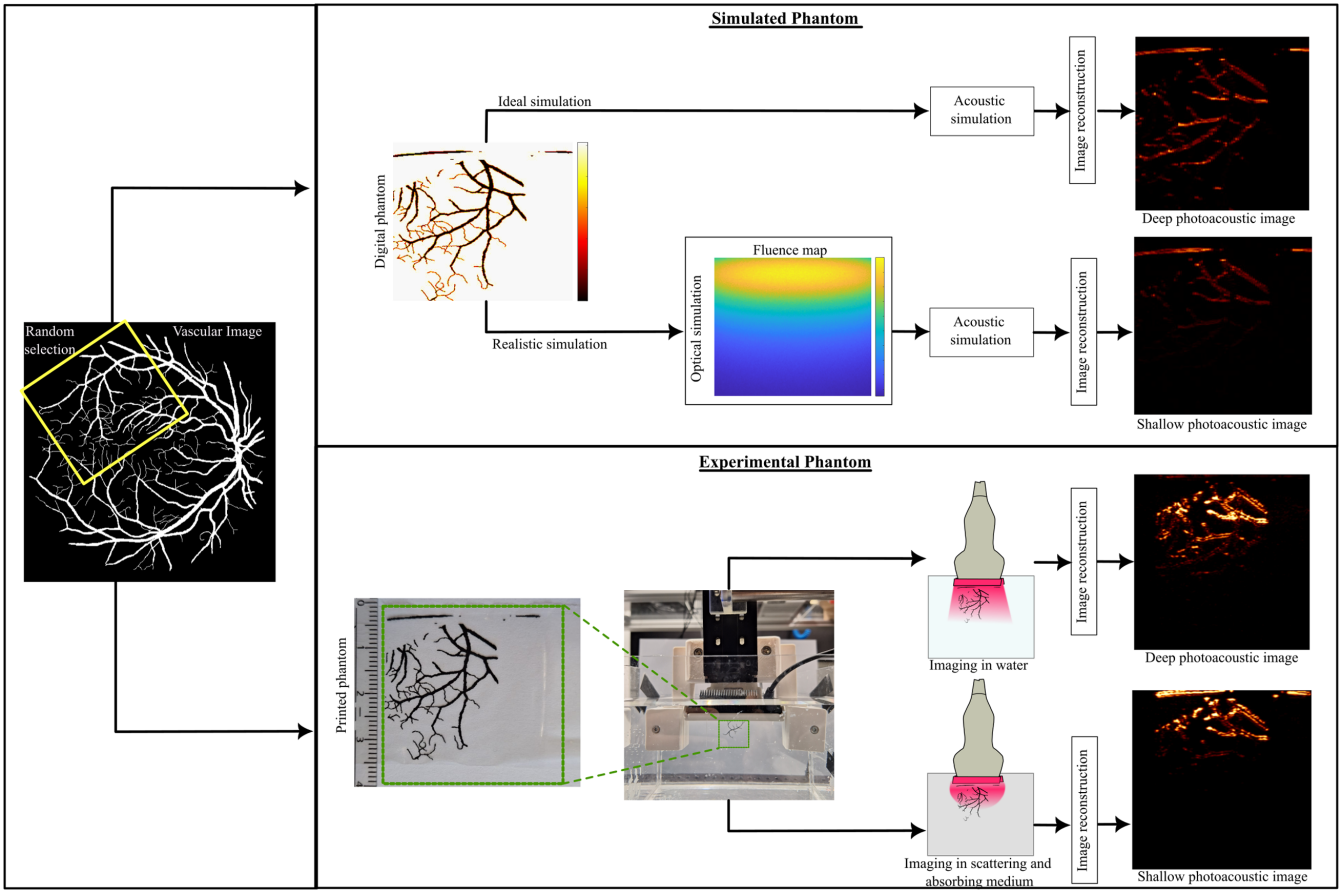
Simulation and experimental twin phantom. The pipeline shows the generation of the simulated and experimental dataset from the digital vascular image. Simulated phantoms consist of acoustic simulation and photoacoustic reconstruction with and without the added light fluence. Experimental phantoms are printed images on polyurethane films imaged in water and an absorbing and scattering medium.

The DRIVE dataset^24^ contains 48 segmented retinal vessel images, each transformed into five variants using augmentation, creating 240 images. From each image, we extracted five non-overlapping regions containing vasculature in different orientations, as shown in Fig. 1. To simulate extracorporeal illumination, a skin-like layer was added to the top of each cropped image. These skin layers were obtained by scanning the forearms of two volunteers at multiple locations using the same imaging system. The average thickness of the skin layer was ∼0.6  mm, which corresponds to about five pixels in the reconstructed images. The NNE dataset^25^ includes 9531 images from 2-photon single-vessel measurements of the mouse SI cortex. Figure 1 outlines the dataset generation pipeline, showing simulation and experimental paths. Initially, vascular images are resized to 40×40  mm. The simulation path involves ideal simulations with uniform light fluence and realistic simulations accounting for fluence decay. In the experimental path, phantoms printed on polyurethane films are imaged in water and tissue-mimicking medium, generating both ideal and realistic PAI. The twin-phantom imaging setup is shown in Fig. S1 in the Supplementary Material.

#### 2.1.1 Optical simulation

We used the LED-based PAI system AcousticX (Cyberdyne Inc., Japan). Light propagation from the integrated LED arrays into tissue was simulated using the GPU-accelerated Monte Carlo eXtreme (MCX) photon transport simulator.^26^ The probe integrates two LED units mounted at a 41.4 deg angle, positioned 0.15 mm in front of the ultrasound transducer and spaced 9.56 mm apart. Each unit measures 50×10  mm and contains four arrays of 144 LED elements (36 per row, 4 rows), emitting at 850 nm with a 120 deg opening angle.

The simulation domain was $55\times 55\times 40\;{\mathrm{mm}}^{3}$, represented by a 744×744×512  voxel grid with a uniform voxel size of 74  μm. Tissue-mimicking optical properties were set as: absorption coefficient $0.01\;{\mathrm{mm}}^{-1}$, reduced scattering coefficient $1\;{\mathrm{mm}}^{-1}$, and anisotropy factor 0.9. Simulations yielded fluence maps in the center plane between the LED arrays. These were normalized and multiplied with digital phantom images to generate initial pressure maps, assuming a constant Grüneisen parameter.

#### 2.1.2 Acoustic simulation and image reconstruction

Acoustic simulations were carried out using the k-Wave toolbox^27^ on a 512×512 grid. Input vascular images were resized and normalized. The corresponding fluence map was cropped and normalized, then multiplied by the image to generate the initial pressure map.

The transducer was modeled as a 128-element linear array with a pitch of 0.315 mm and a center frequency of 7 MHz (bandwidth 80%). Transducer directivity was accounted for via finite element size by summing up sensor data from multiple point sensors. The medium was assumed homogeneous with density $1000\;\mathrm{kg}/m^{3}$ and sound speed 1500  m/s. Additive white noise ranging from 10 to 50 dB (in 10 dB steps) was added to simulate measurement noise. Image reconstruction was performed using a Fourier domain algorithm.^28^

#### 2.1.3 Phantom printing

Digital phantoms were fabricated using polyurethane, chosen for its suitable thermal, optical, and acoustic properties.^29^ The material tolerates the 175°C fuser temperature of the Xerox 7800i printer, with a melting point of 180°C. Its absorption coefficient ranges from 0.001 to $0.005\;{\mathrm{mm}}^{-1}$, and its refractive index (1.41 to 1.58) closely matches soft tissue (1.35 to 1.55).^30^ Acoustic properties include frequency-dependent attenuation of $4\;\mathrm{dB}\;{\mathrm{cm}}^{-1}\;{\mathrm{MHz}}^{-1}$ and impedance of $1.7\times 10^{6}\;\mathrm{Pa}\cdot s\cdot m^{-1}$.^31^ The speed of sound ranges from 1450 to 1730  m/s.^32^

Test vascular phantoms from the DRIVE and NNE datasets were printed on 60  μm thick PCU Protection Cover (Ultrasound B.V., The Netherlands) using black toner, following the method in Ref. 29.

#### 2.1.4 Experimental phantom imaging

Imaging experiments used the handheld AcousticX system.^8^^,^^33^ Phantoms were held in place using a 3D-printed holder with a clamping mechanism to ensure alignment at the center of the transducer array (see Fig. 1). Imaging was conducted in water and in a tissue-mimicking medium. The medium was prepared using 5.5% by volume of a 20% Intralipid solution (Fresenius Kabi, Bad Homburg, Germany) in water for scattering, and Indian ink (Talens, The Netherlands) with a dilution factor of 69,642 for absorption. The resulting optical properties at 850 nm were an absorption coefficient of $0.01\;{\mathrm{mm}}^{-1}$ and a reduced scattering coefficient of $1\;{\mathrm{mm}}^{-1}$, tuned to match the simulation.

### 2.2 Models and Loss Functions

In this section, we detail various generative models and loss functions used in this study. We utilize Pix2Pix GAN, noted for its enhancement of photoacoustic images, as our initial model. Additionally, we explored modified U-Net architectures, including Residual U-Net, and proposed a ViT-based Trans U-Net as generators within the GAN framework. We assess multiple loss functions; MSE, PSNR, SSIM, a combined SSIM and PSNR metric, and GAN loss. Subsequent sections provide detailed implementation, starting with U-Net as the foundational model, followed by descriptions of modifications in the compared models.

#### 2.2.1 Generalized U-Net architecture

**Fig. 2:**
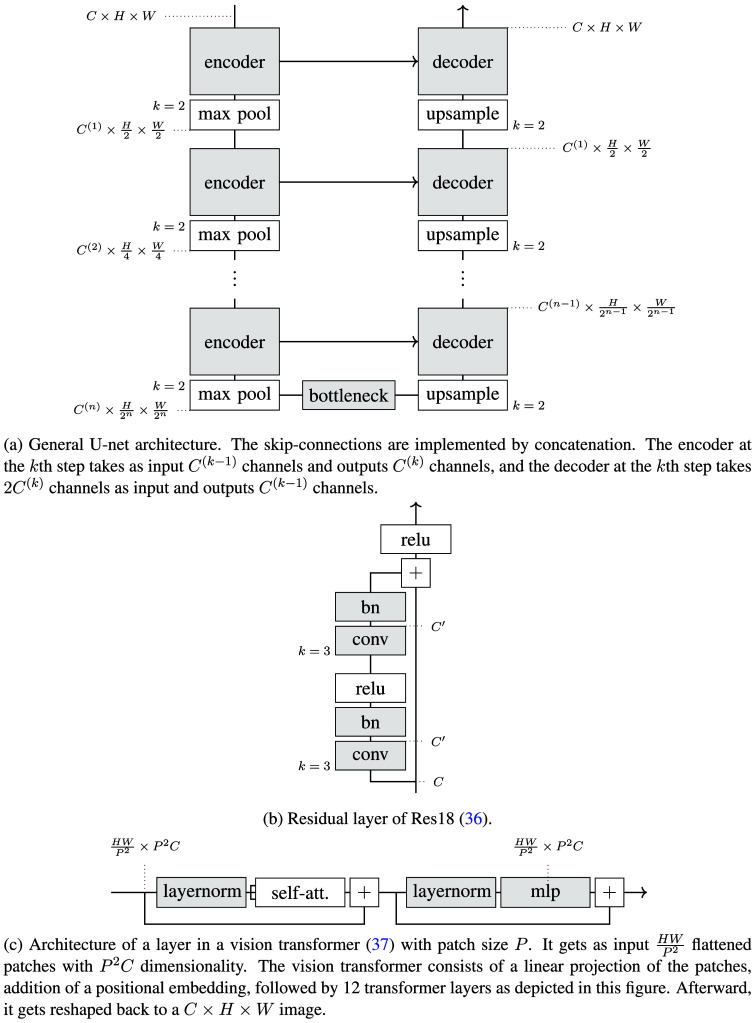
General U-Net architecture, Residual layers of Res18,^34^ and vision transformer. The *bn* block indicates a batchnorm layer and the channel counts at each step are indicated by a dotted line.

A generalized U-Net architecture is illustrated in Fig. 2(a). U-Nets transform images via a two-stage process.^35^ Initially, the input image x is encoded into a high-level latent representation z through a series of encoding steps that incrementally capture information at multiple detail levels. Early layers encode low-level features, such as texture, whereas deeper layers encode high-level features. This encoding phase alternates between encoders and max pooling layers to produce compact latent representations. The second stage involves decoding the latent representation back into an output image y. Each decoder layer takes the output from the previous decoder and receives additional input via skip connections from the corresponding encoder layer, enhancing localization accuracy by combining detailed and contextual information, as depicted in Fig. 2(a).

Differences among U-Net architectures primarily arise from variations in the encoder, decoder, and bottleneck designs, which influence the model’s expressive capability. Additionally, the architecture’s depth and width are controlled by the channel array C, which defines the number of channels at each level. For instance, in this work, we configure C as (64,128,256,512,512,512,512,512), resulting in a latent representation $z\in R^{512}$ from an input image $x\in R^{256\times 256}$, indicating a deep network where each value in the latent representation relates to every input pixel. However, practical applications may require adjustments in C due to rapid increases in parameter count with network size.^35^

#### 2.2.2 Pix2Pix

The first model considered in this work is the generator of the Pix2Pix^36^ architecture, designed initially as a generative adversarial network (GAN). Pix2Pix uses 4×4 convolutional layers in both its encoder and decoder architecture. A ReLU layer precedes the convolutional layer, and a normalization layer comes after it. In the bottom three decoder layers, 50% dropout layers simulate noise to avoid overfitting. This model acts as a baseline for more complex models due to its simplicity.

#### 2.2.3 Residual U-Nets

To overcome the challenge of training very deep convolutional neural networks, we integrated residual layers as proposed by Ref. 34. We defined a learnable mapping $f:R^{C\times H\times W}\to R^{C^{\prime }\times H\times W}$, composed of convolutional layers, normalization layers, and pointwise non-linearities. The weights $W\in R^{C^{\prime }\times C}$ were configured such that the residual layer adds the input directly to the mapped output, as follows,

r(x)=f(x)+Wx. (1)

This configuration allows for an easier propagation of the gradient through the network, simplifying optimization. Furthermore, it makes it possible to initialize at the identity function by initializing f to always output 0, which is generally close to the desired mapping. We utilized ResNet-18 architecture which comprises basic blocks of two convolutional layers with normalization and ReLU activation as depicted in Fig. 2(b).

#### 2.2.4 Trans U-Net

We employed the Trans U-Net^37^ with a ViT^38^ embedded in its bottleneck, aiming to improve high-level image representation for the decoding phase. The architecture of a ViT layer is depicted in Fig. 2(c). Initially, the input image is segmented into patches of dimension C×P×P. These patches are then flattened into vectors and transformed to $\frac{HW}{P^{2}}$ patches of dimension $P^{2}C$. Each patch vector undergoes a linear transformation, followed by the addition of learned positional embeddings. A sequence of 12 transformer layers processes these vectors, as shown in Fig. 2(c), before the decoder reshapes them into a C×H×W format. We augmented the Pix2Pix generator with a ViT to directly compare its performance against traditional Pix2Pix models.

### 2.3 Loss Functions and Image Quality Metrics

To optimize and evaluate the generative models for optical attenuation compensation in PAI, we employed a combination of loss functions and standard image quality metrics. These metrics ensured both robust training and comprehensive performance assessment.

#### 2.3.1 Adversarial loss

Following the Pix2Pix framework,^36^ adversarial loss encourages realistic image generation by introducing a discriminator network $d:R^{C\times H\times W}\to [0,1]$ that learns to discriminate between real outputs of the dataset and fake outputs of the generator network. The generator minimizes a loss combining Binary Cross-Entropy (BCE) and $\ell_{1}$-norm,

ℓGAN(y^,y)=BCE(1,d(y^))+λ‖y−y^‖1, (2)

where y^ is the generated image, y is the target, and λ=50. The $\ell_{1}$ term ensures fidelity to the target, whereas the discriminator term promotes realism. The generator and discriminator are optimized in an alternating fashion, where the discriminator minimizes the following loss,

ℓD(y^,y)=BCE(1,d(y))+BCE(0,d(y^)). (3)

Intuitively, the discriminator wants to assign 1 to the real data point y and 0 to the fake data point from the generator y^. On the other side, the generator wants to “fool” the discriminator by making it output 1 to the generator’s outputs.

#### 2.3.2 Pixel-level loss

We employed MSE loss, defined as

ℓ2(y^,y)=‖y−y^‖22, (4)

to quantify pixel-wise differences, emphasizing larger errors due to the squaring of terms.

#### 2.3.3 Perceptual and structural loss

We incorporated PSNR and SSIM to evaluate perceptual quality and structural fidelity. These metrics were used both as loss functions during training and as evaluation metrics post-training,

ℓPSNR(y^,y)=−PSNR(y^,y), (5)

ℓSSIM(y^,y)=−SSIM(y^,y). (6)

To balance pixel-level accuracy and structural similarity, we combined PSNR and SSIM in a single loss function,

$$\ell_{\mathrm{PSNR}+\mathrm{SSIM}}=\ell_{\mathrm{PSNR}}+\lambda \cdot \ell_{\mathrm{SSIM}},$$ (7)

with λ=30, determined empirically.

### 2.4 Training Details

The simulated photoacoustic images from the DRIVE dataset are split into an 80/20 train-test ratio, with the last 20% of the train split acting as validation data. Thus, 154 images are used for training, 38 for validation, and 48 for testing. Similarly, the photoacoustic images from the NNE dataset are divided into a 67/33 train-test ratio, with the last 20% of the train split serving as validation data. Thus, 5084 images are used for training, 1271 for validation, and 3176 for testing. All these computations are done with a GPU H100 using Adam as an optimizer with a learning rate of ${1e}^{-4}$ and 200 epochs for training the model.^39^ Furthermore, an experimental dataset comprising six test images, three test images from the DRIVE dataset, and three test images from NNE dataset. In this study, the experimental phantoms are only utilized to validate the findings on non-simulated data. The phantoms captured in water serve as ground truth for the images captured in a solution approximating tissue characteristics. The checkpoint for the final model is chosen by maximizing the SSIM score on the validation dataset.

### 2.5 Application in Breast Cancer Imaging

To demonstrate applicability in breast cancer imaging, we have tested the models on both *in vivo* human breast tissue and a small animal tumor model. Acquired images were used to test the trained models.

#### 2.5.1 Imaging in a small animal tumor model

An imaging experiment was conducted on a small animal tumor model, part of a related study.^40^ All animal protocols and experimental procedures were approved by the Institutional Animal Care and Use Committee (IACUC) of the University of Twente. Female SCID mice aged 5 to 7 weeks were procured from Janvier Laboratories and housed under appropriate conditions. The MDA-MB-231 human breast cancer cell line was cultured in high-glucose DMEM supplemented with L-glutamine (GE Healthcare) and 10% fetal bovine serum (FBS). Cells were maintained at 37°C in a humidified incubator with 5% ${\mathrm{CO}}_{2}$. Each mouse was orthotopically injected with $2\times 10^{6}$ MDA-MB-231 cells to induce tumor formation. The endpoint is determined by the tumor volume of $∼1000\;{\mathrm{mm}}^{3}$. The animal was euthanized, and the tumor was imaged immediately.

#### 2.5.2 *In vivo* breast imaging

The AcousticX system was utilized for *in vivo* breast imaging. Imaging was conducted on the left breast of a 31-year-old volunteer with Fitzpatrick skin type IV. Freehand PAI and ultrasound scanning were performed to collect data. Combined ultrasound and photoacoustic images of the breast vasculature were collected and used as test images.

## 3 Results and Discussion

In this section, we first compare fluence (optical attenuation) compensation with Pix2Pix GANs as a baseline generative model, next we show the model predictions on simulated and experimental twin phantoms, followed by a detailed comparison of the generative models and loss function, and finally, we demonstrate its application in *in vivo* imaging.

### 3.1 Generative Model versus Attenuation Compensation

**Fig. 3:**
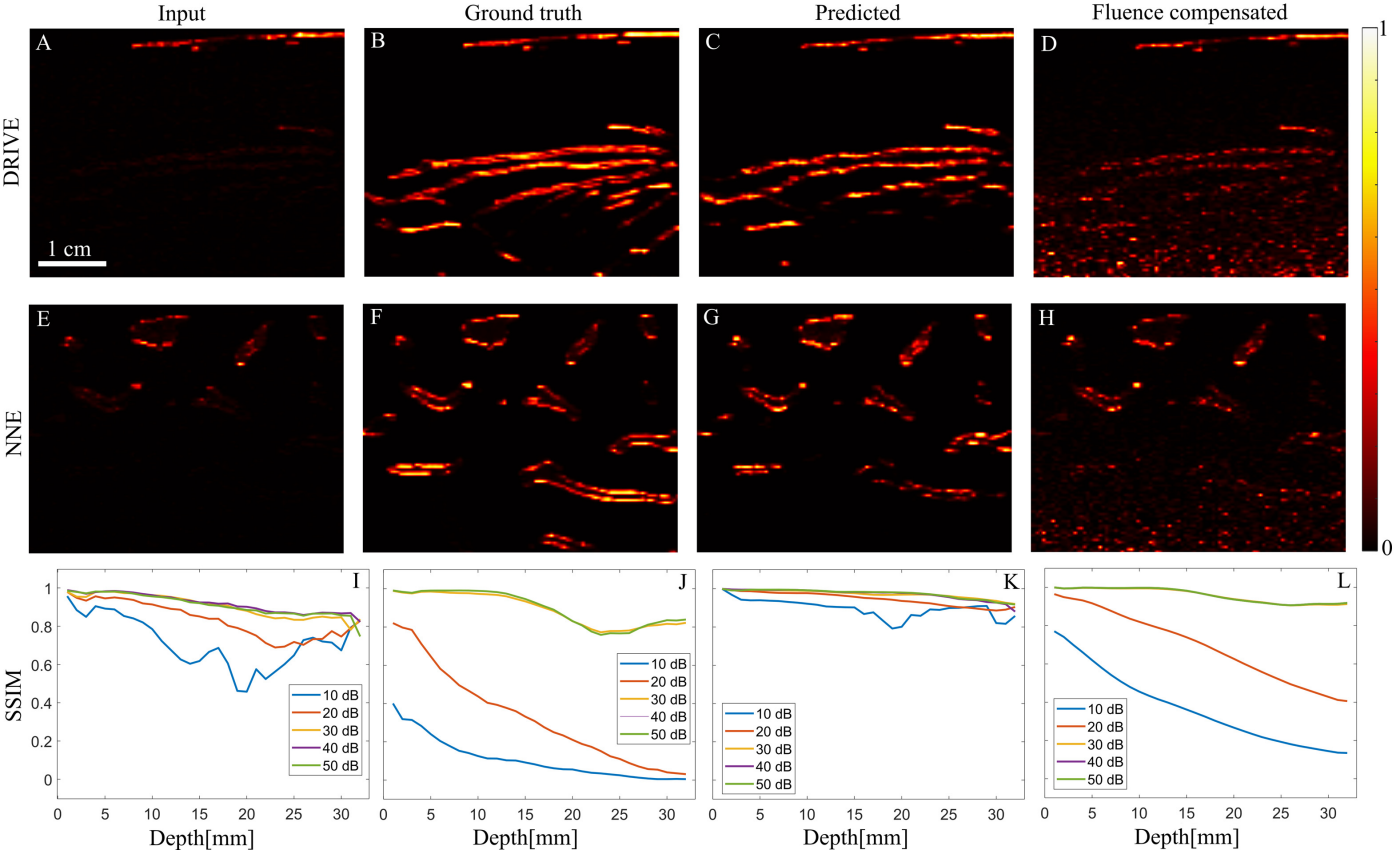
Generative model and fluence compensation comparison. (A–D) Input image, ground truth, Pix2Pix GANs prediction, and fluence compensated image on a test image from the DRIVE dataset. (E–H) Input image, ground truth, Pix2Pix GANs prediction, and fluence-compensated image on a test image from the NNE dataset. (I) Mean SSIM along depth for the Pix2Pix GANs prediction and (J) for the fluence compensation output on the DRIVE dataset test set. (K) Mean SSIM along depth for the Pix2Pix GANs prediction and (L) for the fluence compensation output on the NNE dataset test set.

First, we compare the predictions of a generative model (Pix2Pix GAN) with fluence compensation in PAI. Pix2Pix GANs have previously been employed to enhance photoacoustic images.^41^^,^^42^ Figures 3(a) and 3(e) show the input photoacoustic images from the DRIVE and NNE datasets, respectively. These simulations were performed on an absorbing and scattering medium, resulting in attenuation of fluence with depth. Figures 3(b) and 3(f) display the ground truth photoacoustic images generated using only acoustic simulation and reconstruction, without an optical model, under the assumption of uniform fluence. Figures 3(c) and 3(g) present the predictions from the Pix2Pix GANs model, whereas Figs. 3(d) and 3(h) show the fluence-compensated images. Figures 3(a)–3(h) are obtained at a 30 dB noise level in the simulation. In Figs. 3(i) and 3(j), we compare mean SSIM values computed over depth for the test images of the DRIVE dataset, for the Pix2Pix and fluence compensation methods, respectively. SSIM was computed along the depth direction by extracting image rows corresponding to 2 mm depth intervals and including all columns for each slice. A similar comparison is presented for the NNE dataset in Figs. 3(k) and 3(l).

The results from different noise levels indicate that generative models, such as Pix2Pix GANs, can perform better than the fluence compensation methods by predicting the structure from the noisy input images. Given that the measurement noise also distorts the structures in the image, a direct compensation results in poor structural quality and amplifies noise with depth as evident in Figs. 3(j) and 3(l), where the SSIM value declines with depth at signal-to-noise ratio (SNR) levels of 10 and 20 dB. Although Pix2Pix predictions also show a slight degradation in performance at higher noise levels, they remain comparatively better than the fluence compensation method, as seen in Figs. 3(i)–3(l). These results underscore the potential of deep learning-based methods for compensating for optical attenuation and enhancing structures.

### 3.2 Simulation and Experimental Twin Phantoms

**Fig. 4:**
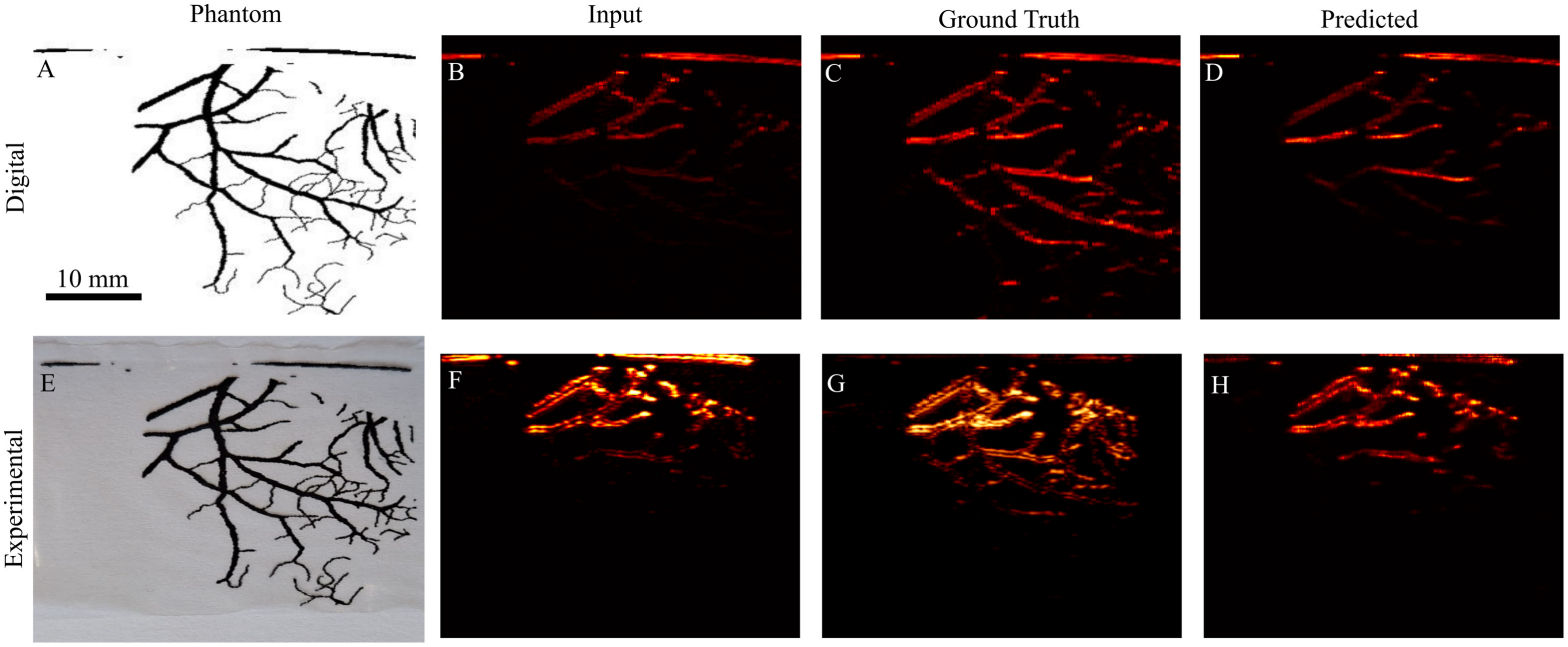
Simulation and experimental photoacoustic twin phantoms: (A) Digital image of the phantom. (B) Simulated photoacoustic image with fluence in a tissue-mimicking medium. (C) Ground truth photoacoustic image assuming uniform fluence. (D) Pix2Pix prediction with (B) as input. (E) Printed phantom on a polyurethane film. (F) PAI in a tissue-mimicking medium. (G) PAI in water. (H) Pix2Pix prediction with (F) as input.

We present the use of simulation and experimental twin phantoms to find the practical capability of predictive models. Figure 4(a) shows the digital phantom. Figure 4(b) displays the simulated photoacoustic image incorporating attenuation due to fluence, whereas Fig. 4(c) presents the simulation considering uniform illumination. Figure 4(d) illustrates the Pix2Pix prediction using Fig. 4(b) as input. Figure 4(e) shows the printed phantom on a polyurethane film. Figure 4(f) depicts imaging in an absorbing and scattering medium, and Fig. 4(g) shows imaging in water. Finally, Fig. 4(h) demonstrates the Pix2Pix prediction using Fig. 4(f) as input.

The results demonstrate that we can obtain experimental phantoms with structural ground truth information to compare with model predictions. This approach facilitates testing of deep learning models using similar simulated and experimental data. Furthermore, the ground truth information available in the experimental data enables verification of the prediction accuracy. However, discrepancies between the simulated and experimental results suggest that accurate modeling of all experimental parameters is necessary. Although we have considered the transducer and medium parameters here, the modeling is based on ideal system specifications and lacks fine-tuning based on actual measurements. The key takeaway is the availability of structural information to evaluate how the model performs on experimental data. It can be observed that Pix2Pix predictions enhance imaging depth compared with the input in both simulated and experimental phantoms. However, when compared with the ground truth images, the depth recovery in the Pix2Pix predictions is limited. The vascular structures that appear enhanced are largely the same ones already faintly visible in the input images at lower pixel intensities, rather than new structures revealed at greater depth. Pix2Pix predictions enhanced low photoacoustic signal structures with low background noise. Given that the Pix2Pix prediction only provided marginal improvement at deeper structures, we further compare Res18 U-Net and the proposed Trans U-Net and loss functions to identify the most suitable model for this task.

### 3.3 Comparison of Generative Models and Loss Functions

**Fig. 5:**
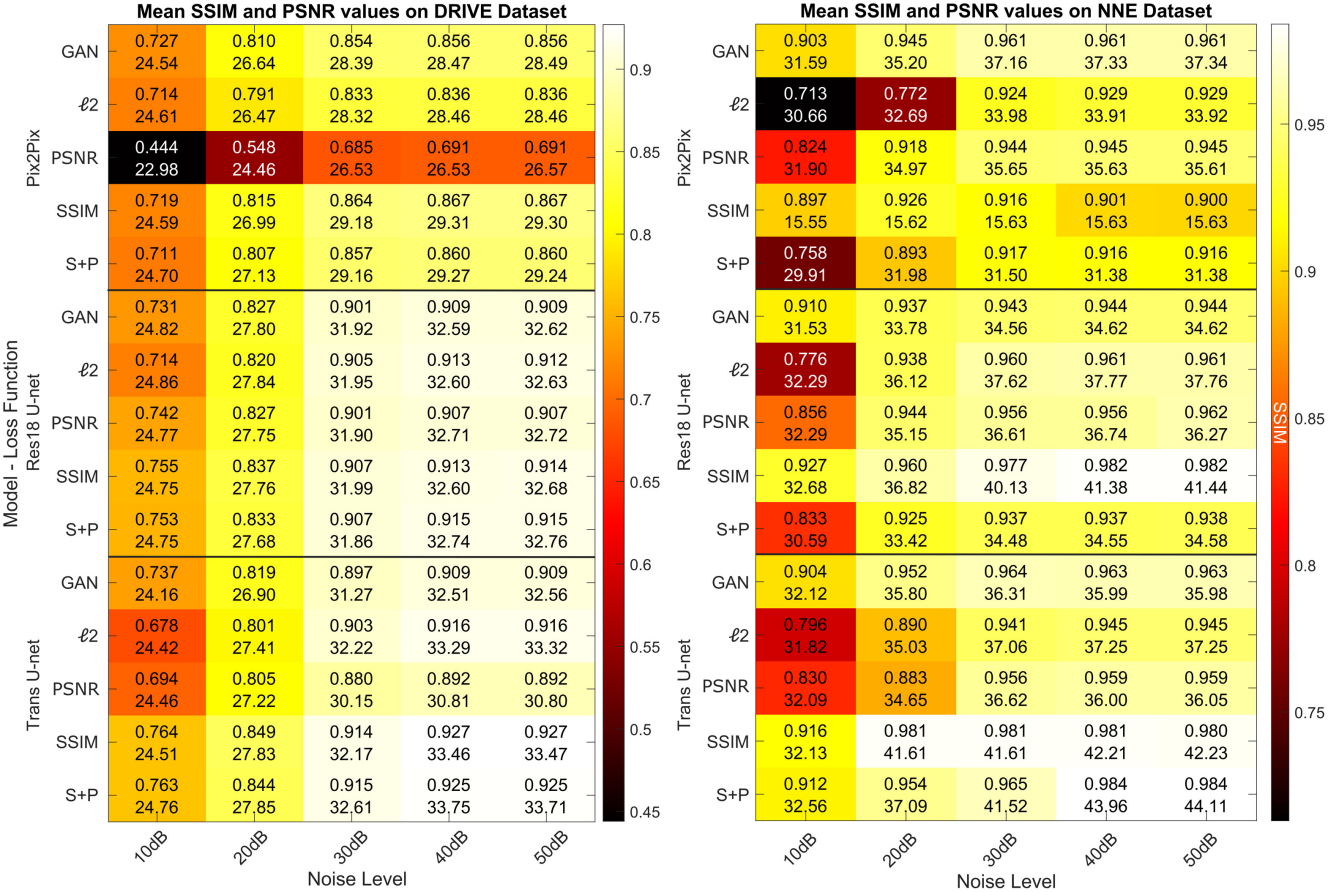
The table shows SSIM and PSNR with heatmaps based on SSIM value, illustrate the performance of three models, Pix2Pix, Res18 U-Net, and Trans U-Net, evaluated using various loss functions: GAN, l2 (MSE), PSNR, SSIM, and a combined SSIM + PSNR (S + P). Performance is assessed across noise levels ranging from 10 dB to 50 dB. The table on the left presents results for models trained on the DRIVE dataset, whereas the table on the right shows results for models trained on the NNE dataset.

In Fig. 5, we compare the performance of the three models, Pix2Pix, Res18 U-Net and Trans U-Net on input images from noise levels ranging from 10 to 50 dB. We also compare different loss functions. The performance is compared in terms of image quality metrics SSIM and PSNR listed together. The heatmap shows SSIM to provide a visual impression of model performance. Figure 5 left, shows the performance of models on the DRIVE dataset, and Fig. 5 right corresponds to the NNE dataset.

When trained with the pre-specified SSIM + PSNR loss, Trans U-Net is statistically superior to Res18-UNet and Pix2Pix for SSIM and PSNR on both datasets across noise levels, except for a single non-significant PSNR comparison on DRIVE at 10 dB. Under alternative loss functions, performance is mixed and in some settings other models are comparable or better, as detailed in Table S1 in the Supplementary Material. This suggests that the proposed transformer-based architecture is more robust in handling fluence attenuation and noise in photoacoustic images. Among the loss functions tested, SSIM and combined SSIM + PSNR yield the highest SSIM and PSNR values, indicating their effectiveness in preserving structural and perceptual quality. Notably, the SSIM loss function performs best on the DRIVE dataset, whereas the combined SSIM + PSNR achieves the highest scores on the NNE dataset, suggesting that the optimal loss function may be dataset-dependent. The NNE dataset consistently shows higher SSIM and PSNR values across all models and loss functions compared with DRIVE, implying that NNE images may be less complex or contain more consistent features that are easier to reconstruct. Overall, the combination of Trans U-Net with loss functions SSIM or SSIM + PSNR provides the most reliable performance, and the results highlight the importance of tailoring model-loss configurations to specific dataset characteristics.

#### 3.3.1 Statistical significance

We have performed a statistical paired t-test to show which model is superior in terms of PSNR and SSIM for the different loss function compared for different models in various noise conditions. For comparisons, we have compared the best performing model with the second best model.

Trans U-Net trained with SSIM + PSNR shows significant improvements in most comparisons on both datasets, with all NNE comparisons significant and one non-significant PSNR comparison on DRIVE at 10 dB. The best results were obtained using the combined SSIM + PSNR loss. These improvements were statistically significant (p<0.05) in most cases (Table 1). Detailed per-loss and per-noise-level comparisons are provided in Table S1 in the Supplementary Material.

**Table 1:** Statistical significance of Trans U-Net compared with Pix2Pix and Res18 U-Net across different metrics, noise levels, and two datasets. Significance determined by paired t-test (p<0.05) for the S + P loss function.

Dataset	Noise (dB)	Trans U-Net versus Pix2Pix		Trans U-Net versus Res18 U-Net	
		PSNR	SSIM	PSNR	SSIM
DRIVE Dataset	10	No	Yes	No	Yes
	20	Yes	Yes	Yes	Yes
	30	Yes	Yes	Yes	Yes
	40	Yes	Yes	Yes	Yes
	50	Yes	Yes	Yes	Yes
NNE 10 dataset	10	Yes	Yes	Yes	Yes
	20	Yes	Yes	Yes	Yes
	30	Yes	Yes	Yes	Yes
	40	Yes	Yes	Yes	Yes
	50	Yes	Yes	Yes	Yes

#### 3.3.2 Testing on a vascular printed phantom

**Fig. 6:**
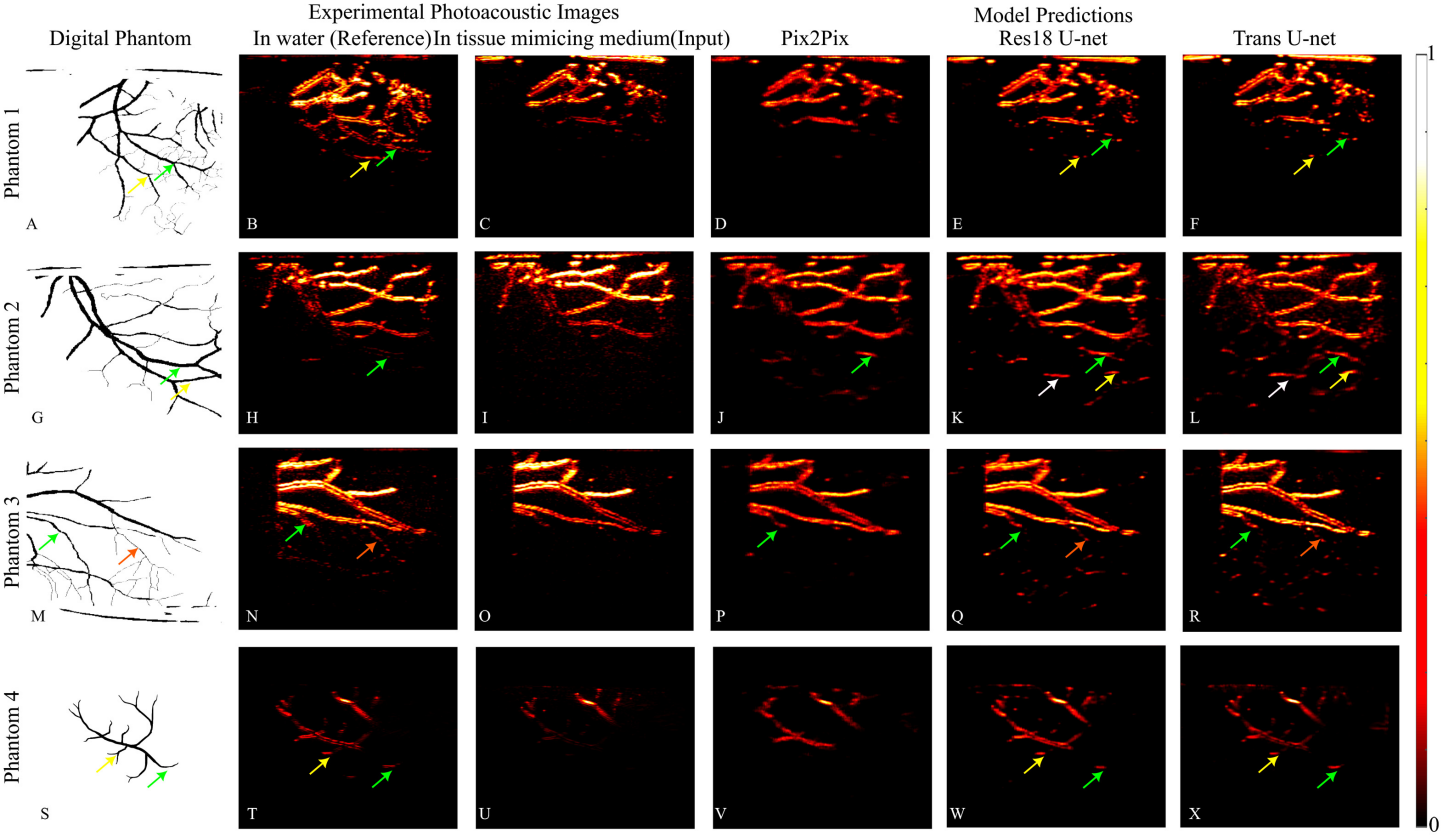
Imaging of Printed Vascular Phantoms. (A, G, M, S) show the ground truth vascular phantoms. Among these, (A, G, M) are from the test dataset, whereas (S) represents a mouse brain vascular phantom not included in the dataset.^27^ (B, H, N, T) display the phantoms printed in water. (C, I, O, U) show the printed phantoms embedded in tissue-mimicking medium, illustrating depth-dependent attenuation. (D, J, P, V) present predictions from the Pix2Pix model. (E, K, Q, W) show predictions from the Res18 U-Net model. (F, L, R, X) depict predictions from the Trans U-Net model. Colored arrows highlight vascular structures accurately predicted by the models.

We tested trained models on a printed vascular phantom. Figure 6 shows the phantoms and the model prediction. Ground truth vascular structures are shown in Figs. 6(a), 6(g), 6(m), and 6(s). The corresponding physical phantoms printed in water are presented in Figs. 6(b), 6(h), 6(n), and 6(t), as a reference in a non-scattering medium to visualize deeper structures, whereas Figs. 6(c), 6(i), 6(o), and 6(u) display the same phantoms embedded in a tissue-mimicking medium, demonstrating depth-dependent photoacoustic signal attenuation. Model predictions are shown in the subsequent rows: Pix2Pix outputs are illustrated in Figs. 6(d), 6(j), 6(p), and 6(v), Res18 U-Net predictions in Figs. 6(e), 6(k), 6(q), and 6(w), and Trans U-Net predictions in Figs. 6(f), 6(l), 6(r), and 6(x). The colored arrows in ground truth digital images and in the model predictions show structures that are enhanced by the model. Visually, it can be observed that Trans U-Net enhanced most structures compared with the digital ground truth image, followed by Res18 U-Net. Pix2Pix predictions resulted in the least depth enhancement. We computed the relative SNR as 10log_10_() of the ratio between the mean squared value of the reference image patch and the MSE between the predicted image patch and the reference patch obtained in water. Quantitative evaluation of the relative SNR for marked vascular structures revealed the models’ performance. Pix2Pix achieved an average SNR of 2.3±1.5  dB, indicating limited enhancement and high variability. In contrast, Res18 U-Net and Trans U-Net demonstrated substantially higher SNRs of 5.4±1.3 and 5.3±1.2  dB, respectively, reflecting improved signal clarity and consistency. For the printed phantoms, Res18 U-Net and the proposed Trans U-Net showed comparable performance in terms of SNR.

### 3.4 *In Vivo* Tumor Imaging

**Fig. 7:**
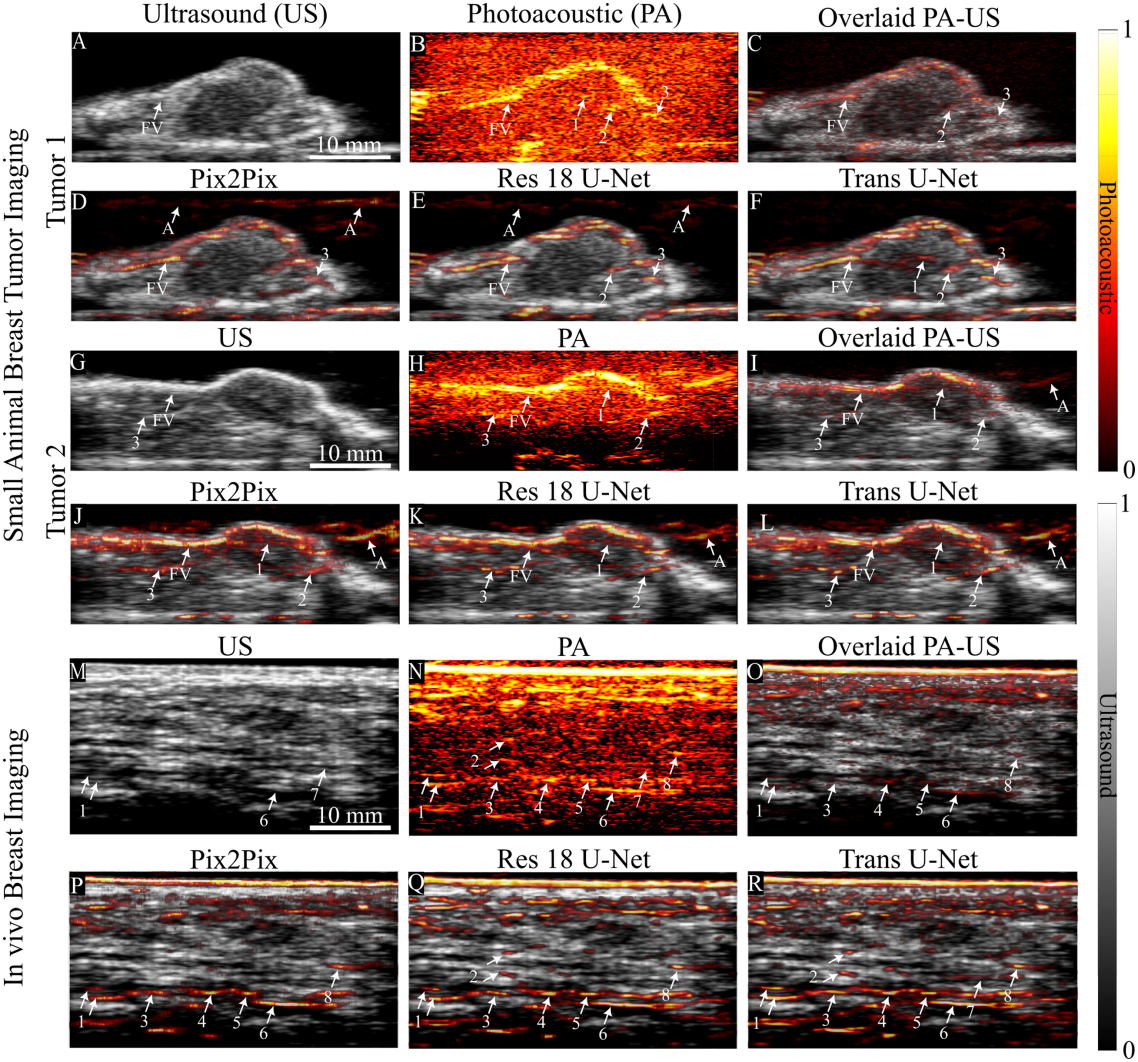
*In vivo* testing. (A–L) Model testing on small animal breast tumor images, (M–R) Model testing on human breast imaging. (A, G, and M) are ultrasound images. (B, H, and N) Photoacoustic images in log scale. (C, I, O) Overlaid photoacoustic images on ultrasound images. (D, J, and P) Prediction using Pix2Pix. (E, K, and Q) Prediction using Res18 and (F, L, and R) prediction using Trans U-Net. (Arrows with FV and A indicate feeding blood vessel and artefacts, respectively).

We evaluated Pix2Pix, Res18 U-Net, and Trans U-Net on *in vivo* murine breast tumor models and on *in vivo* human breast images to demonstrate applicability to breast cancer imaging. Figures 7(a)–7(l) show two small-animal tumors and Figs. 7(m)–7(r) show human breast imaging. Figures 7(a), 7(g), and 7(m) are ultrasound. Figures 7(b), 7(h), and 7(n) are the corresponding photoacoustic images in log scale. Figures 7(c), 7(i), and 7(o) are photoacoustic overlaid on ultrasound. Figures 7(d), 7(j), and 7(p) are Pix2Pix predictions. Figures 7(e), 7(k), and 7(q) are Res18 U-Net, and Figs. 7(f), 7(l), and 7(r) are Trans U-Net. The arrows locate vascular features that are faint but present in the log-scale input photoacoustic images, and, where visible, hypoechoic tracks on ultrasound that often co-localize with blood vessels. These markings anchor the qualitative assessment to signals already present in the raw data rather than to structures created by the networks. Feeding blood vessels (FV) and their branching into the tumor can be observed in the enhanced images in both tumors.

Across both murine tumors and human breast, Trans U-Net produced the clearest enhancement of the vascular signals than Pix2Pix, and generally stronger contrast than Res18 U-Net. Some examples of Trans U-Net enhancing vascular structures better than Res18 and Pix2Pix are arrow marked 1 in Figs. 7(b) and 7(f), and arrow marked 7 in Figs. 7(n) and 7(r). Some false predictions are also visible. Limited-view, low-SNR traces can be overconnected into vessel-like strands, and reconstruction artefacts outside tissue can be modified into vascular-like patterns by all three models, most prominently by Pix2Pix, but also Res18 and Trans U-Net. We therefore restrict interpretation to regions where the enhanced predictions correspond to signals visible in the log-scale image and, when available, to hypoechoic tracks on ultrasound. These *in vivo* observations are consistent with quantitative trends in simulated test sets, with the Trans U-Net trained with SSIM + PSNR loss achieving the highest attenuation compensation and denoising.

## 4 Limitations and Future Work

The experimental phantoms were used only for testing the model. Training the model on experimental data could improve performance, but this requires a scalable method to automate the imaging of printed films and generate larger datasets. The phantoms, constrained by limited thickness, do not fully replicate the 3D nature of blood vessels. Our focus was on structural recovery rather than signal quantification. We have previously shown that varying film opacity can simulate different absorption levels.^29^ Future work will investigate the quantification of recovered signals for applications such as oxygen saturation.

*In vivo*, there is no absolute ground truth. To avoid over-claiming, we anchored our interpretation to two forms of evidence visible in the photoacoustic signal in the log-scale image; second, spatial co-localization and ultrasound hypoechoic tracks. Using this conservative approach, Trans U-Net most consistently enhanced vessel-like structures that were already detectable. At the same time, we explicitly show false predictions, including the conversion of reconstruction artefacts into vessel-like patterns and the overconnection of weak, limited-view traces. These observations caution against treating the network output as a vascular prediction *in vivo* and motivate future validation with an independent modality such as Doppler or contrast-enhanced ultrasound and inclusion of artifact exemplars during training. Our simulation and phantom experiments, where ground truth is available, show that Trans U-Net is the most accurate model by SSIM and PSNR and has a slight advantage over Res18 U-Net on printed phantoms and *in vivo* scenarios.

We also observed variability in model performance across datasets. Although we compared a state-of-the-art Transformer-based model, this variability remains evident. In contrast to common observations that U-Nets perform better on smaller datasets, in our study the transformer-based model outperformed both Res18 U-Net and Pix2Pix, likely reflecting the combined effect of dataset quality and the ability of transformers to capture long-range dependencies. Although the model was trained exclusively on simulated data, which allowed systematic validation with ground-truth access, we acknowledge that this may limit its direct generalization to experimental and *in vivo* conditions; future work will focus on fine-tuning and validating the framework on empirical datasets using transfer learning and domain adaptation strategies. Developing a universal model^43^ that is robust across multiple PAI systems and tissue types remains a key goal for advancing PAI. Extending this approach using newer generative models, such as Trans U-Net, is a promising future direction.

## 5 Conclusion

We addressed the coupled problem of fluence attenuation and denoising using generative models. We proposed a ViT-based Trans U-Net and compared it with state-of-the-art models such as Pix2Pix and Residual U-Net (Res18). On simulated datasets, Trans U-Net combined with the SSIM + PSNR loss function showed superior performance in recovering vascular structures and denoising photoacoustic images. In printed experimental phantoms, however, Res18 U-Net and Trans U-Net showed comparable performance, whereas Pix2Pix performed relatively worse. The SSIM + PSNR loss function emerged as the most effective, balancing structural fidelity and noise suppression. *In vivo* experiments further validated the practical utility of these models, with Trans U-Net notably enhancing vascular visualization in small animal tumor imaging. These findings highlight the potential of generative models, particularly Trans U-Net, to improve image quality in PAI and support more reliable and informative biomedical applications.

## Supplementary Material

10.1117/1.JBO.30.11.116004.s01

## Data Availability

The code and dataset for the models are available at https://github.com/navchetan-awasthi/pai-reconstruction.
