# Supplementary material for: Transformer-based optical attenuation compensation and denoising in photoacoustic imaging
Source: J Biomed Opt. 2025 Nov 26;30(11):116004. doi: 10.1117/1.JBO.30.11.116004 (PMC12654947; doi:10.1117/1.JBO.30.11.116004)
Supplement: Supplementary file 1 [file JBO_030_116004_SD001.pdf]

Supplementary Material: Transformer-Based Optical Attenuation Compensation and Denoising in Photoacoustic Imaging

Cristian Perez Jensen[a], **Navchetan Awasthi**[a,b], **Kalloor Joseph Francis** [c]
[a]Faculty of Science, Mathematics and Computer Science, Informatics Institute, University of Amsterdam, Amsterdam, 1090 GH, The Netherlands
[b]Department of Biomedical Engineering and Physics, Amsterdam UMC, Amsterdam, 1081 HV, The Netherlands
[c]Erasmus MC, Cardiovascular Institute, Department of Cardiology, Biomedical Engineering, Rotterdam, The Netherlands

1 Twin phantom imaging setup

Figure 1 shows the photoacoustic probe with a linear array and LED array together with a 3D printed phantom holder to place the printed phantom in the imaging plane.

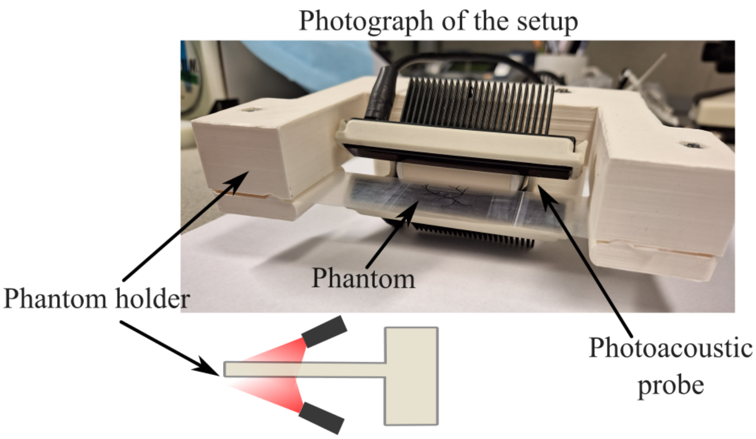

Fig 1: Imaging setup for twin phantom imaging.

2 Comparison of models

The table shows comparison of the proposed Trans U-Net with other models.

Table 1: Statistical Significance Summary Across Models

Dataset	Loss	Noise	Metric	Comparison	p-value	Better Model
DRIVE	GAN	10dB	PSNR	Trans U-Net vs Pix2Pix	$1.18e^{-07}$	Pix2Pix
DRIVE	GAN	10dB	PSNR	Trans U-Net vs Res18 U-Net	$5.49e^{-14}$	Res18 U-Net
DRIVE	GAN	10dB	SSIM	Trans U-Net vs Pix2Pix	$4.74e^{-03}$	Trans U-Net
DRIVE	GAN	10dB	SSIM	Trans U-Net vs Res18 U-Net	$8.28e^{-03}$	Trans U-Net
DRIVE	GAN	20dB	PSNR	Trans U-Net vs Pix2Pix	$2.97e^{-02}$	Trans U-Net
DRIVE	GAN	20dB	PSNR	Trans U-Net vs Res18 U-Net	$8.54e^{-14}$	Res18 U-Net
DRIVE	GAN	20dB	SSIM	Trans U-Net vs Pix2Pix	$8.61e^{-05}$	Trans U-Net
DRIVE	GAN	20dB	SSIM	Trans U-Net vs Res18 U-Net	$8.93e^{-06}$	Res18 U-Net
DRIVE	GAN	30dB	PSNR	Trans U-Net vs Pix2Pix	$1.11e^{-22}$	Trans U-Net
DRIVE	GAN	30dB	PSNR	Trans U-Net vs Res18 U-Net	$9.22e^{-09}$	Res18 U-Net
DRIVE	GAN	30dB	SSIM	Trans U-Net vs Pix2Pix	$1.01e^{-20}$	Trans U-Net
DRIVE	GAN	30dB	SSIM	Trans U-Net vs Res18 U-Net	$7.17e^{-06}$	Res18 U-Net
DRIVE	GAN	40dB	PSNR	Trans U-Net vs Pix2Pix	$7.62e^{-28}$	Trans U-Net
DRIVE	GAN	40dB	PSNR	Trans U-Net vs Res18 U-Net	$4.06e^{-01}$	Res18 U-Net
DRIVE	GAN	40dB	SSIM	Trans U-Net vs Pix2Pix	$9.66e^{-24}$	Trans U-Net
DRIVE	GAN	40dB	SSIM	Trans U-Net vs Res18 U-Net	$8.84e^{-01}$	Res18 U-Net
DRIVE	GAN	50dB	PSNR	Trans U-Net vs Pix2Pix	$4.36e^{-28}$	Trans U-Net

Dataset	Loss	Noise	Metric	Comparison	p-value	Better Model
DRIVE	GAN	50dB	PSNR	Trans U-Net vs Res18 U-Net	$4.78e^{-01}$	Res18 U-Net
DRIVE	GAN	50dB	SSIM	Trans U-Net vs Pix2Pix	$1.45e^{-23}$	Trans U-Net
DRIVE	GAN	50dB	SSIM	Trans U-Net vs Res18 U-Net	$9.55e^{-01}$	Res18 U-Net
DRIVE	MSE	10dB	PSNR	Trans U-Net vs Pix2Pix	$3.71e^{-03}$	Pix2Pix
DRIVE	MSE	10dB	PSNR	Trans U-Net vs Res18 U-Net	$6.38e^{-11}$	Res18 U-Net
DRIVE	MSE	10dB	SSIM	Trans U-Net vs Pix2Pix	$8.61e^{-18}$	Pix2Pix
DRIVE	MSE	10dB	SSIM	Trans U-Net vs Res18 U-Net	$1.20e^{-16}$	Res18 U-Net
DRIVE	MSE	20dB	PSNR	Trans U-Net vs Pix2Pix	$6.85e^{-12}$	Trans U-Net
DRIVE	MSE	20dB	PSNR	Trans U-Net vs Res18 U-Net	$1.35e^{-07}$	Res18 U-Net
DRIVE	MSE	20dB	SSIM	Trans U-Net vs Pix2Pix	$8.00e^{-04}$	Trans U-Net
DRIVE	MSE	20dB	SSIM	Trans U-Net vs Res18 U-Net	$9.86e^{-11}$	Res18 U-Net
DRIVE	MSE	30dB	PSNR	Trans U-Net vs Pix2Pix	$1.30e^{-28}$	Trans U-Net
DRIVE	MSE	30dB	PSNR	Trans U-Net vs Res18 U-Net	$9.26e^{-03}$	Trans U-Net
DRIVE	MSE	30dB	SSIM	Trans U-Net vs Pix2Pix	$9.11e^{-28}$	Trans U-Net
DRIVE	MSE	30dB	SSIM	Trans U-Net vs Res18 U-Net	$4.54e^{-02}$	Res18 U-Net
DRIVE	MSE	40dB	PSNR	Trans U-Net vs Pix2Pix	$4.85e^{-29}$	Trans U-Net
DRIVE	MSE	40dB	PSNR	Trans U-Net vs Res18 U-Net	$4.00e^{-09}$	Trans U-Net
DRIVE	MSE	40dB	SSIM	Trans U-Net vs Pix2Pix	$4.88e^{-31}$	Trans U-Net
DRIVE	MSE	40dB	SSIM	Trans U-Net vs Res18 U-Net	$5.51e^{-05}$	Trans U-Net
DRIVE	MSE	50dB	PSNR	Trans U-Net vs Pix2Pix	$2.59e^{-28}$	Trans U-Net
DRIVE	MSE	50dB	PSNR	Trans U-Net vs Res18 U-Net	$7.35e^{-10}$	Trans U-Net
DRIVE	MSE	50dB	SSIM	Trans U-Net vs Pix2Pix	$1.24e^{-30}$	Trans U-Net
DRIVE	MSE	50dB	SSIM	Trans U-Net vs Res18 U-Net	$2.92e^{-05}$	Trans U-Net
DRIVE	PSNR	10dB	PSNR	Trans U-Net vs Pix2Pix	$9.64e^{-11}$	Trans U-Net
DRIVE	PSNR	10dB	PSNR	Trans U-Net vs Res18 U-Net	$8.35e^{-11}$	Res18 U-Net
DRIVE	PSNR	10dB	SSIM	Trans U-Net vs Pix2Pix	$2.98e^{-33}$	Trans U-Net
DRIVE	PSNR	10dB	SSIM	Trans U-Net vs Res18 U-Net	$2.55e^{-25}$	Res18 U-Net
DRIVE	PSNR	20dB	PSNR	Trans U-Net vs Pix2Pix	$8.37e^{-15}$	Trans U-Net
DRIVE	PSNR	20dB	PSNR	Trans U-Net vs Res18 U-Net	$1.00e^{-09}$	Res18 U-Net
DRIVE	PSNR	20dB	SSIM	Trans U-Net vs Pix2Pix	$5.15e^{-36}$	Trans U-Net
DRIVE	PSNR	20dB	SSIM	Trans U-Net vs Res18 U-Net	$4.87e^{-13}$	Res18 U-Net
DRIVE	PSNR	30dB	PSNR	Trans U-Net vs Pix2Pix	$8.24e^{-18}$	Trans U-Net
DRIVE	PSNR	30dB	PSNR	Trans U-Net vs Res18 U-Net	$1.15e^{-19}$	Res18 U-Net
DRIVE	PSNR	30dB	SSIM	Trans U-Net vs Pix2Pix	$6.23e^{-31}$	Trans U-Net
DRIVE	PSNR	30dB	SSIM	Trans U-Net vs Res18 U-Net	$2.21e^{-17}$	Res18 U-Net
DRIVE	PSNR	40dB	PSNR	Trans U-Net vs Pix2Pix	$6.12e^{-20}$	Trans U-Net
DRIVE	PSNR	40dB	PSNR	Trans U-Net vs Res18 U-Net	$9.97e^{-22}$	Res18 U-Net
DRIVE	PSNR	40dB	SSIM	Trans U-Net vs Pix2Pix	$1.50e^{-30}$	Trans U-Net
DRIVE	PSNR	40dB	SSIM	Trans U-Net vs Res18 U-Net	$1.12e^{-11}$	Res18 U-Net
DRIVE	PSNR	50dB	PSNR	Trans U-Net vs Pix2Pix	$1.64e^{-19}$	Trans U-Net
DRIVE	PSNR	50dB	PSNR	Trans U-Net vs Res18 U-Net	$4.79e^{-21}$	Res18 U-Net
DRIVE	PSNR	50dB	SSIM	Trans U-Net vs Pix2Pix	$2.04e^{-30}$	Trans U-Net
DRIVE	PSNR	50dB	SSIM	Trans U-Net vs Res18 U-Net	$8.61e^{-12}$	Res18 U-Net
DRIVE	SSIM	10dB	PSNR	Trans U-Net vs Pix2Pix	$3.56e^{-02}$	Pix2Pix
DRIVE	SSIM	10dB	PSNR	Trans U-Net vs Res18 U-Net	$4.46e^{-08}$	Res18 U-Net
DRIVE	SSIM	10dB	SSIM	Trans U-Net vs Pix2Pix	$2.32e^{-24}$	Trans U-Net
DRIVE	SSIM	10dB	SSIM	Trans U-Net vs Res18 U-Net	$3.18e^{-09}$	Trans U-Net
DRIVE	SSIM	20dB	PSNR	Trans U-Net vs Pix2Pix	$3.67e^{-11}$	Trans U-Net
DRIVE	SSIM	20dB	PSNR	Trans U-Net vs Res18 U-Net	$1.79e^{-01}$	Trans U-Net
DRIVE	SSIM	20dB	SSIM	Trans U-Net vs Pix2Pix	$1.32e^{-21}$	Trans U-Net
DRIVE	SSIM	20dB	SSIM	Trans U-Net vs Res18 U-Net	$2.52e^{-15}$	Trans U-Net
DRIVE	SSIM	30dB	PSNR	Trans U-Net vs Pix2Pix	$2.22e^{-21}$	Trans U-Net
DRIVE	SSIM	30dB	PSNR	Trans U-Net vs Res18 U-Net	$6.16e^{-02}$	Trans U-Net
DRIVE	SSIM	30dB	SSIM	Trans U-Net vs Pix2Pix	$2.05e^{-23}$	Trans U-Net
DRIVE	SSIM	30dB	SSIM	Trans U-Net vs Res18 U-Net	$1.09e^{-07}$	Trans U-Net
DRIVE	SSIM	40dB	PSNR	Trans U-Net vs Pix2Pix	$4.39e^{-27}$	Trans U-Net
DRIVE	SSIM	40dB	PSNR	Trans U-Net vs Res18 U-Net	$5.41e^{-13}$	Trans U-Net
DRIVE	SSIM	40dB	SSIM	Trans U-Net vs Pix2Pix	$3.47e^{-26}$	Trans U-Net
DRIVE	SSIM	40dB	SSIM	Trans U-Net vs Res18 U-Net	$6.86e^{-19}$	Trans U-Net
DRIVE	SSIM	50dB	PSNR	Trans U-Net vs Pix2Pix	$4.39e^{-26}$	Trans U-Net
DRIVE	SSIM	50dB	PSNR	Trans U-Net vs Res18 U-Net	$4.86e^{-11}$	Trans U-Net
DRIVE	SSIM	50dB	SSIM	Trans U-Net vs Pix2Pix	$3.72e^{-26}$	Trans U-Net
DRIVE	SSIM	50dB	SSIM	Trans U-Net vs Res18 U-Net	$1.05e^{-18}$	Trans U-Net
DRIVE	SSIM+PSNR	10dB	PSNR	Trans U-Net vs Pix2Pix	$1.50e^{-01}$	Trans U-Net
DRIVE	SSIM+PSNR	10dB	PSNR	Trans U-Net vs Res18 U-Net	$5.61e^{-01}$	Trans U-Net

Dataset	Loss	Noise	Metric	Comparison	p-value	Better Model
DRIVE	SSIM+PSNR	10dB	SSIM	Trans U-Net vs Pix2Pix	$2.32e^{-20}$	Trans U-Net
DRIVE	SSIM+PSNR	10dB	SSIM	Trans U-Net vs Res18 U-Net	$5.10e^{-03}$	Trans U-Net
DRIVE	SSIM+PSNR	20dB	PSNR	Trans U-Net vs Pix2Pix	$3.50e^{-08}$	Trans U-Net
DRIVE	SSIM+PSNR	20dB	PSNR	Trans U-Net vs Res18 U-Net	$2.06e^{-03}$	Trans U-Net
DRIVE	SSIM+PSNR	20dB	SSIM	Trans U-Net vs Pix2Pix	$6.75e^{-23}$	Trans U-Net
DRIVE	SSIM+PSNR	20dB	SSIM	Trans U-Net vs Res18 U-Net	$3.46e^{-14}$	Trans U-Net
DRIVE	SSIM+PSNR	30dB	PSNR	Trans U-Net vs Pix2Pix	$1.32e^{-23}$	Trans U-Net
DRIVE	SSIM+PSNR	30dB	PSNR	Trans U-Net vs Res18 U-Net	$1.01e^{-10}$	Trans U-Net
DRIVE	SSIM+PSNR	30dB	SSIM	Trans U-Net vs Pix2Pix	$1.94e^{-28}$	Trans U-Net
DRIVE	SSIM+PSNR	30dB	SSIM	Trans U-Net vs Res18 U-Net	$1.52e^{-12}$	Trans U-Net
DRIVE	SSIM+PSNR	40dB	PSNR	Trans U-Net vs Pix2Pix	$6.03e^{-27}$	Trans U-Net
DRIVE	SSIM+PSNR	40dB	PSNR	Trans U-Net vs Res18 U-Net	$1.30e^{-15}$	Trans U-Net
DRIVE	SSIM+PSNR	40dB	SSIM	Trans U-Net vs Pix2Pix	$1.02e^{-27}$	Trans U-Net
DRIVE	SSIM+PSNR	40dB	SSIM	Trans U-Net vs Res18 U-Net	$9.81e^{-19}$	Trans U-Net
DRIVE	SSIM+PSNR	50dB	PSNR	Trans U-Net vs Pix2Pix	$2.27e^{-26}$	Trans U-Net
DRIVE	SSIM+PSNR	50dB	PSNR	Trans U-Net vs Res18 U-Net	$8.78e^{-16}$	Trans U-Net
DRIVE	SSIM+PSNR	50dB	SSIM	Trans U-Net vs Pix2Pix	$1.65e^{-27}$	Trans U-Net
DRIVE	SSIM+PSNR	50dB	SSIM	Trans U-Net vs Res18 U-Net	$7.09e^{-18}$	Trans U-Net
NNE	GAN	10dB	PSNR	Trans U-Net vs Pix2Pix	0.00	Trans U-Net
NNE	GAN	10dB	PSNR	Trans U-Net vs Res18 U-Net	$2.11e^{-185}$	Trans U-Net
NNE	GAN	10dB	SSIM	Trans U-Net vs Pix2Pix	$2.75e^{-01}$	Trans U-Net
NNE	GAN	10dB	SSIM	Trans U-Net vs Res18 U-Net	$2.95e^{-19}$	Res18 U-Net
NNE	GAN	20dB	PSNR	Trans U-Net vs Pix2Pix	$6.14e^{-185}$	Trans U-Net
NNE	GAN	20dB	PSNR	Trans U-Net vs Res18 U-Net	0.00	Trans U-Net
NNE	GAN	20dB	SSIM	Trans U-Net vs Pix2Pix	0.00	Trans U-Net
NNE	GAN	20dB	SSIM	Trans U-Net vs Res18 U-Net	0.00	Trans U-Net
NNE	GAN	30dB	PSNR	Trans U-Net vs Pix2Pix	$6.90e^{-124}$	Pix2Pix
NNE	GAN	30dB	PSNR	Trans U-Net vs Res18 U-Net	0.00	Trans U-Net
NNE	GAN	30dB	SSIM	Trans U-Net vs Pix2Pix	$4.91e^{-220}$	Trans U-Net
NNE	GAN	30dB	SSIM	Trans U-Net vs Res18 U-Net	0.00	Trans U-Net
NNE	GAN	40dB	PSNR	Trans U-Net vs Pix2Pix	$8.85e^{-273}$	Pix2Pix
NNE	GAN	40dB	PSNR	Trans U-Net vs Res18 U-Net	$6.14e^{-223}$	Trans U-Net
NNE	GAN	40dB	SSIM	Trans U-Net vs Pix2Pix	$2.24e^{-162}$	Trans U-Net
NNE	GAN	40dB	SSIM	Trans U-Net vs Res18 U-Net	0.00	Trans U-Net
NNE	GAN	50dB	PSNR	Trans U-Net vs Pix2Pix	$2.32e^{-278}$	Pix2Pix
NNE	GAN	50dB	PSNR	Trans U-Net vs Res18 U-Net	$4.80e^{-221}$	Trans U-Net
NNE	GAN	50dB	SSIM	Trans U-Net vs Pix2Pix	$4.99e^{-179}$	Trans U-Net
NNE	GAN	50dB	SSIM	Trans U-Net vs Res18 U-Net	0.00	Trans U-Net
NNE	MSE	10dB	PSNR	Trans U-Net vs Pix2Pix	0.00	Trans U-Net
NNE	MSE	10dB	PSNR	Trans U-Net vs Res18 U-Net	$4.93e^{-122}$	Res18 U-Net
NNE	MSE	10dB	SSIM	Trans U-Net vs Pix2Pix	0.00	Trans U-Net
NNE	MSE	10dB	SSIM	Trans U-Net vs Res18 U-Net	$4.75e^{-50}$	Trans U-Net
NNE	MSE	20dB	PSNR	Trans U-Net vs Pix2Pix	0.00	Trans U-Net
NNE	MSE	20dB	PSNR	Trans U-Net vs Res18 U-Net	0.00	Res18 U-Net
NNE	MSE	20dB	SSIM	Trans U-Net vs Pix2Pix	0.00	Trans U-Net
NNE	MSE	20dB	SSIM	Trans U-Net vs Res18 U-Net	0.00	Res18 U-Net
NNE	MSE	30dB	PSNR	Trans U-Net vs Pix2Pix	0.00	Trans U-Net
NNE	MSE	30dB	PSNR	Trans U-Net vs Res18 U-Net	$8.27e^{-161}$	Res18 U-Net
NNE	MSE	30dB	SSIM	Trans U-Net vs Pix2Pix	0.00	Trans U-Net
NNE	MSE	30dB	SSIM	Trans U-Net vs Res18 U-Net	0.00	Res18 U-Net
NNE	MSE	40dB	PSNR	Trans U-Net vs Pix2Pix	0.00	Trans U-Net
NNE	MSE	40dB	PSNR	Trans U-Net vs Res18 U-Net	$3.04e^{-127}$	Res18 U-Net
NNE	MSE	40dB	SSIM	Trans U-Net vs Pix2Pix	0.00	Trans U-Net
NNE	MSE	40dB	SSIM	Trans U-Net vs Res18 U-Net	0.00	Res18 U-Net
NNE	MSE	50dB	PSNR	Trans U-Net vs Pix2Pix	0.00	Trans U-Net
NNE	MSE	50dB	PSNR	Trans U-Net vs Res18 U-Net	$1.05e^{-125}$	Res18 U-Net
NNE	MSE	50dB	SSIM	Trans U-Net vs Pix2Pix	0.00	Trans U-Net
NNE	MSE	50dB	SSIM	Trans U-Net vs Res18 U-Net	0.00	Res18 U-Net
NNE	PSNR	10dB	PSNR	Trans U-Net vs Pix2Pix	$3.85e^{-12}$	Trans U-Net
NNE	PSNR	10dB	PSNR	Trans U-Net vs Res18 U-Net	$7.67e^{-17}$	Res18 U-Net
NNE	PSNR	10dB	SSIM	Trans U-Net vs Pix2Pix	$1.04e^{-04}$	Trans U-Net
NNE	PSNR	10dB	SSIM	Trans U-Net vs Res18 U-Net	$1.81e^{-148}$	Res18 U-Net
NNE	PSNR	20dB	PSNR	Trans U-Net vs Pix2Pix	$9.94e^{-12}$	Pix2Pix
NNE	PSNR	20dB	PSNR	Trans U-Net vs Res18 U-Net	$8.58e^{-21}$	Res18 U-Net
NNE	PSNR	20dB	SSIM	Trans U-Net vs Pix2Pix	$1.81e^{-192}$	Pix2Pix
NNE	PSNR	20dB	SSIM	Trans U-Net vs Res18 U-Net	0.00	Res18 U-Net
NNE	PSNR	30dB	PSNR	Trans U-Net vs Pix2Pix	$1.57e^{-264}$	Trans U-Net
NNE	PSNR	30dB	PSNR	Trans U-Net vs Res18 U-Net	$7.21e^{-01}$	Trans U-Net
NNE	PSNR	30dB	SSIM	Trans U-Net vs Pix2Pix	$2.21e^{-154}$	Trans U-Net

Dataset	Loss	Noise	Metric	Comparison	p-value	Better Model
NNE	PSNR	30dB	SSIM	Trans U-Net vs Res18 U-Net	$3.29e^{-01}$	Res18 U-Net
NNE	PSNR	40dB	PSNR	Trans U-Net vs Pix2Pix	$5.28e^{-75}$	Trans U-Net
NNE	PSNR	40dB	PSNR	Trans U-Net vs Res18 U-Net	$2.82e^{-75}$	Res18 U-Net
NNE	PSNR	40dB	SSIM	Trans U-Net vs Pix2Pix	0.00	Trans U-Net
NNE	PSNR	40dB	SSIM	Trans U-Net vs Res18 U-Net	$8.80e^{-146}$	Trans U-Net
NNE	PSNR	50dB	PSNR	Trans U-Net vs Pix2Pix	$4.88e^{-61}$	Trans U-Net
NNE	PSNR	50dB	PSNR	Trans U-Net vs Res18 U-Net	$5.80e^{-89}$	Res18 U-Net
NNE	PSNR	50dB	SSIM	Trans U-Net vs Pix2Pix	0.00	Trans U-Net
NNE	PSNR	50dB	SSIM	Trans U-Net vs Res18 U-Net	$3.85e^{-122}$	Trans U-Net
NNE	SSIM	10dB	PSNR	Trans U-Net vs Pix2Pix	0.00	Trans U-Net
NNE	SSIM	10dB	PSNR	Trans U-Net vs Res18 U-Net	$4.87e^{-258}$	Res18 U-Net
NNE	SSIM	10dB	SSIM	Trans U-Net vs Pix2Pix	$9.87e^{-107}$	Trans U-Net
NNE	SSIM	10dB	SSIM	Trans U-Net vs Res18 U-Net	$2.48e^{-34}$	Res18 U-Net
NNE	SSIM	20dB	PSNR	Trans U-Net vs Pix2Pix	0.00	Trans U-Net
NNE	SSIM	20dB	PSNR	Trans U-Net vs Res18 U-Net	$2.30e^{-17}$	Trans U-Net
NNE	SSIM	20dB	SSIM	Trans U-Net vs Pix2Pix	0.00	Trans U-Net
NNE	SSIM	20dB	SSIM	Trans U-Net vs Res18 U-Net	$1.26e^{-41}$	Res18 U-Net
NNE	SSIM	30dB	PSNR	Trans U-Net vs Pix2Pix	0.00	Trans U-Net
NNE	SSIM	30dB	PSNR	Trans U-Net vs Res18 U-Net	0.00	Trans U-Net
NNE	SSIM	30dB	SSIM	Trans U-Net vs Pix2Pix	0.00	Trans U-Net
NNE	SSIM	30dB	SSIM	Trans U-Net vs Res18 U-Net	$2.91e^{-241}$	Trans U-Net
NNE	SSIM	40dB	PSNR	Trans U-Net vs Pix2Pix	0.00	Trans U-Net
NNE	SSIM	40dB	PSNR	Trans U-Net vs Res18 U-Net	$3.84e^{-296}$	Trans U-Net
NNE	SSIM	40dB	SSIM	Trans U-Net vs Pix2Pix	0.00	Trans U-Net
NNE	SSIM	40dB	SSIM	Trans U-Net vs Res18 U-Net	$4.17e^{-24}$	Res18 U-Net
NNE	SSIM	50dB	PSNR	Trans U-Net vs Pix2Pix	0.00	Trans U-Net
NNE	SSIM	50dB	PSNR	Trans U-Net vs Res18 U-Net	$1.34e^{-270}$	Trans U-Net
NNE	SSIM	50dB	SSIM	Trans U-Net vs Pix2Pix	0.00	Trans U-Net
NNE	SSIM	50dB	SSIM	Trans U-Net vs Res18 U-Net	$6.73e^{-51}$	Res18 U-Net
NNE	SSIM+PSNR	10dB	PSNR	Trans U-Net vs Pix2Pix	0.00	Trans U-Net
NNE	SSIM+PSNR	10dB	PSNR	Trans U-Net vs Res18 U-Net	0.00	Trans U-Net
NNE	SSIM+PSNR	10dB	SSIM	Trans U-Net vs Pix2Pix	0.00	Trans U-Net
NNE	SSIM+PSNR	10dB	SSIM	Trans U-Net vs Res18 U-Net	0.00	Trans U-Net
NNE	SSIM+PSNR	20dB	PSNR	Trans U-Net vs Pix2Pix	0.00	Trans U-Net
NNE	SSIM+PSNR	20dB	PSNR	Trans U-Net vs Res18 U-Net	0.00	Trans U-Net
NNE	SSIM+PSNR	20dB	SSIM	Trans U-Net vs Pix2Pix	0.00	Trans U-Net
NNE	SSIM+PSNR	20dB	SSIM	Trans U-Net vs Res18 U-Net	0.00	Trans U-Net
NNE	SSIM+PSNR	30dB	PSNR	Trans U-Net vs Pix2Pix	0.00	Trans U-Net
NNE	SSIM+PSNR	30dB	PSNR	Trans U-Net vs Res18 U-Net	0.00	Trans U-Net
NNE	SSIM+PSNR	30dB	SSIM	Trans U-Net vs Pix2Pix	0.00	Trans U-Net
NNE	SSIM+PSNR	30dB	SSIM	Trans U-Net vs Res18 U-Net	0.00	Trans U-Net
NNE	SSIM+PSNR	40dB	PSNR	Trans U-Net vs Pix2Pix	0.00	Trans U-Net
NNE	SSIM+PSNR	40dB	PSNR	Trans U-Net vs Res18 U-Net	0.00	Trans U-Net
NNE	SSIM+PSNR	40dB	SSIM	Trans U-Net vs Pix2Pix	0.00	Trans U-Net
NNE	SSIM+PSNR	40dB	SSIM	Trans U-Net vs Res18 U-Net	0.00	Trans U-Net
NNE	SSIM+PSNR	50dB	PSNR	Trans U-Net vs Pix2Pix	0.00	Trans U-Net
NNE	SSIM+PSNR	50dB	PSNR	Trans U-Net vs Res18 U-Net	0.00	Trans U-Net
NNE	SSIM+PSNR	50dB	SSIM	Trans U-Net vs Pix2Pix	0.00	Trans U-Net
NNE	SSIM+PSNR	50dB	SSIM	Trans U-Net vs Res18 U-Net	0.00	Trans U-Net
